# Altered Fecal Small RNA Profiles in Colorectal Cancer Reflect Gut Microbiome Composition in Stool Samples

**DOI:** 10.1128/mSystems.00289-19

**Published:** 2019-09-17

**Authors:** Sonia Tarallo, Giulio Ferrero, Gaetano Gallo, Antonio Francavilla, Giuseppe Clerico, Alberto Realis Luc, Paolo Manghi, Andrew Maltez Thomas, Paolo Vineis, Nicola Segata, Barbara Pardini, Alessio Naccarati, Francesca Cordero

**Affiliations:** a Italian Institute for Genomic Medicine (IIGM), Turin, Italy; b Department of Computer Science, University of Turin, Turin, Italy; c Department of Surgical and Medical Sciences, University of Catanzaro, Catanzaro, Italy; d Department of Colorectal Surgery, Clinica S. Rita, Vercelli, Italy; e Department CIBIO, University of Trento, Trento, Italy; f Imperial College, London, United Kingdom; g Department of Medical Sciences, University of Turin, Turin, Italy; h Department of Molecular Biology of Cancer, Institute of Experimental Medicine, Prague, Czech Republic; Luxembourg Centre for Systems Biomedicine

**Keywords:** gut microbiome, human stool samples, small RNAs, microRNAs

## Abstract

Dysbiotic configurations of the human gut microbiota have been linked to colorectal cancer (CRC). Human small noncoding RNAs are also implicated in CRC, and recent findings suggest that their release in the gut lumen contributes to shape the gut microbiota. Bacterial small RNAs (bsRNAs) may also play a role in carcinogenesis, but their role has been less extensively explored. Here, we performed small RNA and shotgun sequencing on 80 stool specimens from patients with CRC or with adenomas and from healthy subjects collected in a cross-sectional study to evaluate their combined use as a predictive tool for disease detection. We observed considerable overlap and a correlation between metagenomic and bsRNA quantitative taxonomic profiles obtained from the two approaches. We identified a combined predictive signature composed of 32 features from human and microbial small RNAs and DNA-based microbiome able to accurately classify CRC samples separately from healthy and adenoma samples (area under the curve [AUC] = 0.87). In the present study, we report evidence that host-microbiome dysbiosis in CRC can also be observed by examination of altered small RNA stool profiles. Integrated analyses of the microbiome and small RNAs in the human stool may provide insights for designing more-accurate tools for diagnostic purposes.

**IMPORTANCE** The characteristics of microbial small RNA transcription are largely unknown, while it is of primary importance for a better identification of molecules with functional activities in the gut niche under both healthy and disease conditions. By performing combined analyses of metagenomic and small RNA sequencing (sRNA-Seq) data, we characterized both the human and microbial small RNA contents of stool samples from healthy individuals and from patients with colorectal carcinoma or adenoma. With the integrative analyses of metagenomic and sRNA-Seq data, we identified a human and microbial small RNA signature which can be used to improve diagnosis of the disease. Our analysis of human and gut microbiome small RNA expression is relevant to generation of the first hypotheses about the potential molecular interactions occurring in the gut of CRC patients, and it can be the basis for further mechanistic studies and clinical tests.

## Abstract (precis)

The characteristics of microbial small RNA transcription are largely unknown, while it is of primary importance for a better identification of molecules with functional activities in the gut niche under both healthy and disease conditions. By performing combined analyses of metagenomic and small RNA sequencing (sRNA-Seq) data, we characterized both the human and microbial small RNA contents of stool samples from healthy individuals and from patients with colorectal carcinoma or adenoma. With the integrative analyses of metagenomic and sRNA-Seq data, we identified a human and microbial small RNA signature which can be used to improve diagnosis of the disease. Our analysis of human and gut microbiome small RNA expression is relevant to generation of the first hypotheses about the potential molecular interactions occurring in the gut of CRC patients, and it can be the basis for further mechanistic studies and clinical tests.

## INTRODUCTION

A strong link between gut microbial dysbiosis and colorectal cancer (CRC) has been repeatedly reported in the last years (1, 2). The term “oncobiome” has been used to describe this close relationship between the microbiome and cancer. In particular, the oncobiome relates to microbes that can cause cell transformation by affecting genome stability, resistance to cell death, and proliferative signaling (3). Specific microbial signatures have been associated with CRC (2, 4–6). Besides bacteria such as Fusobacterium nucleatum, toxinogenic and pathological strains of normally commensal species such as Escherichia coli and Bacteroides fragilis also seem to be involved in CRC onset and development (1, 2, 7).

Importantly, host and gut microbiome interactions are mediated by proteins, metabolites, and small RNAs (sRNAs), including Homo sapiens microRNAs (hsa-miRNAs) or other human small noncoding RNAs (hsa-sncRNAs) (8). Liu and colleagues demonstrated that the alteration of intestinal Mus musculus miRNA (mmu-miRNA) synthesis promotes gut dysbiosis that could be restored by fecal transplantation of mmu-miRNAs (9). In the same study, the authors observed that specific hsa-miRNAs can be taken up by E. coli and F. nucleatum bacteria, regulating the expression of microbial genes and affecting their growth. Different studies have reported an alteration of hsa-miRNA expression promoted by perturbation of the gut microbiome or by specific microbial species (8, 10, 11). In the context of CRC, previous studies correlated the gut microbiome composition with a signature of hsa-miRNAs differentially expressed between CRC tissues and adjacent colonic mucosa (12, 13). Furthermore, the incubation of CRC cell lines with F. nucleatum induced the expression of hsa-miR-21 by activation of the Toll-like receptor 4 (TLR4) signaling pathway (14). Interestingly, F. nucleatum sRNAs, as well as Epstein-Barr virus miRNAs, were upregulated in CRC tissues compared to adjacent normal mucosa (15) supporting the idea of a role of a reciprocal interaction between host and gut microbiome sRNAs.

Microbial sRNAs play a critical role in microbial gene regulatory networks (16), but thus far, their involvement in host-microbiome interactions has been less extensively explored. In addition to the human sRNAs which can affect microbial gene expression (9), these molecules may contribute to an interkingdom RNA regulatory network by modulating microbial and human gene expression levels (17, 18). Many microbial RNA sequences can be detected in transcriptomic experiments performed on human samples (19, 20). However, their characterization remains challenging because the short length of sRNA transcripts (50 to 200 nucleotides [nt]) hampers their identification and quantification, especially when the microbial genomic sequence, usable as a reference, is not available. In this sense, the integration of small RNA sequencing (sRNA-Seq) and whole-metagenome sequencing profiles (21) could make the identification of microbial sRNAs more reliable and informative in searches of complementary cancer biomarkers.

In the present study, we sequenced stool samples collected from 80 subjects (24 healthy individuals, 27 adenomas, and 29 CRC) in a hospital-based case-control study. By integrating sRNA-Seq and metagenomic analyses, we provided evidence of extensive overlap of microbial populations detected by the two approaches with the identification of abundant microbial sRNAs in carcinoma patients. Finally, we reported a combination of DNA and RNA signatures which is able to accurately classify CRC patients with respect to precancerous lesions and healthy controls.

## RESULTS

### Differences in fecal microbiome composition among healthy, adenoma, and CRC patients.

The study included 80 subjects who provided stool samples before undergoing colonoscopy. DNA and RNA fractions were extracted from each fecal sample for metagenomic and sRNA-Seq (5, 22) (see Materials and Methods).

The taxonomic characterization of the microbiome revealed 427 bacterial species belonging to 11 phyla (see Table S1A [https://doi.org/10.6084/m9.figshare.8078630]). The most abundant bacterial species in both healthy and adenoma samples was Faecalibacterium prausnitzii (8.9% and 6.4%, respectively) while Alistipes putredinis was the most abundant species across the CRC samples (4.8%).

**FIG 1:**
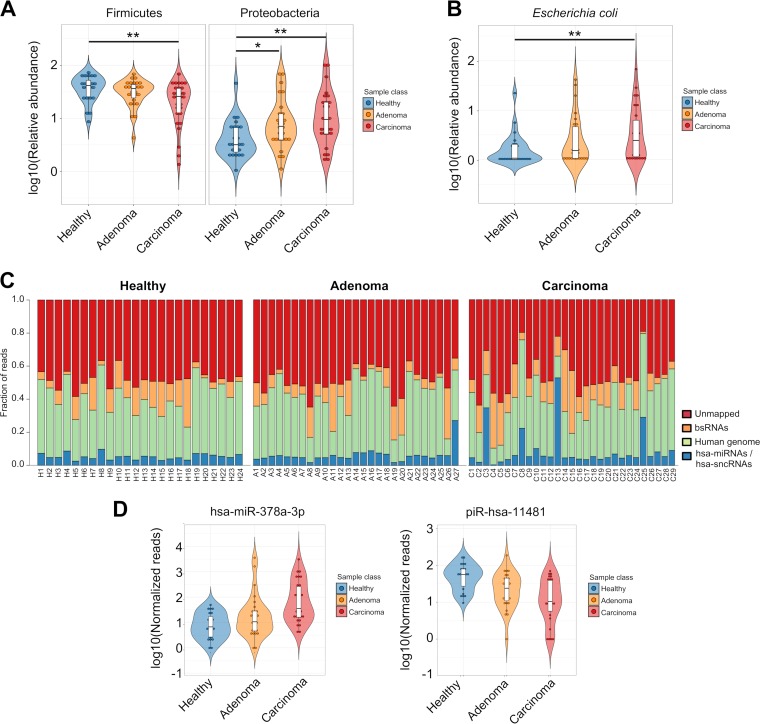
(A) Violin plots reporting the relative abundances of differentially abundant bacterial phyla among healthy, adenoma, and carcinoma groups from metagenomic data analysis. *P* values were computed using the Wilcoxon rank sum test and adjusted using the Benjamini-Hochberg method. **, adjusted *P* value < 0.01; *, adjusted *P* value < 0.05. (B) Violin plots reporting the relative abundances of E. coli from metagenomic data analysis. *P* values were computed using the Wilcoxon rank sum test and adjusted using the Benjamini-Hochberg method. **, adjusted *P* value < 0.01. (C) Stacked bar plot reporting the fraction of sRNA-Seq reads assigned to hsa-miRNAs or hsa-sncRNAs (blue), human genome (green), or bsRNAs (orange) or that were not mapped (red). (D) Violin plots reporting the expression levels for the most significant differentially expressed hsa-miRNA (left) and hsa-sncRNA (right) between the healthy and carcinoma groups (DESeq2 adjusted *P* value < 0.05).

At the phylum level, the abundances of *Proteobacteria* and *Verrucomicrobia* in adenoma patients were significantly different from those seen in both the healthy and cancer groups. In particular, *Verrucomicrobia* showed the highest abundance whereas *Proteobacteria* showed intermediate abundance in the adenoma group (see Table S1A). *Firmicutes* was the most significantly abundant phylum in the carcinoma group compared with both the healthy and adenoma groups (Fig. 1A; see also Table S1A).

At the species level, a significant increase in the levels of E. coli DNA was observed in the healthy samples compared to the adenoma and CRC samples (adjusted *P* value, <0.01) (Fig. 1B) whereas the levels of Bifidobacterium catenulatum DNA and Eubacterium eligens DNA were decreased (adjusted *P* value, <0.01) (see Table S1B).

### The spectrum of fecal human sncRNAs.

The analysis of human and microbial sRNA expression in stool samples was performed by applying a modified sRNA-Seq analysis pipeline developed by our group (22, 23). Starting from an average of 9.3 million single-end reads per sample, a median of 5.3% of the reads were assigned to human sRNA annotations (Fig. 1C; see also Table S2 [https://doi.org/10.6084/m9.figshare.8078633]).

Collectively, 141 hsa-miRNAs and 973 hsa-sncRNAs were associated with at least one sample group (with a median of at least 20 reads). Overall, comparing healthy to adenoma subjects, adenoma to carcinoma subjects, and healthy to carcinoma subjects, 14, 54, and 87 differentially expressed hsa-miRNAs were identified, respectively (see Fig. S1A [https://doi.org/10.6084/m9.figshare.8081003] and Table S3A [https://doi.org/10.6084/m9.figshare.8078627]). Furthermore, 70, 47, and 4 hsa-sncRNAs (other than hsa-miRNAs) were dysregulated in the comparisons described above, respectively (see Fig. S1B and Table S3B). Among the differentially expressed sncRNAs, tRNAs were the most highly represented biotype (55.4%), followed by Piwi-interacting RNAs (piRNAs) (31.3%).

The hsa-miRNA and hsa-sncRNA found to be most significantly differently expressed in the comparison between cancer and healthy groups were hsa-miR-378a-3p (adjusted *P* value, <0.0001) and hsa-piR-11481 (adjusted *P* value, <0.02), respectively (Fig. 1D).

Focusing on hsa-miRNAs only, two annotations (hsa-miR-200b-3p and hsa-miR-6738-5p) were detected as significantly dysregulated in all the comparisons (see Fig. S1C). The hsa-miR-200b-3p expression levels progressively increased whereas those of hsa-miR-6738-5p gradually decreased going from the healthy to the adenoma to the CRC group (see Fig. S1D).

In addition to the two hsa-miRNAs that were found significantly altered in all the comparisons, in the adenoma group, we also identified hsa-miR-30c-5p, which was the hsa-miRNA most highly differentially expressed in this group compared with the other two. In contrast, 43 differentially expressed hsa-miRNAs characterized the CRC group. A total of 5 and 33 altered hsa-miRNAs showed expression trends of gradual increases and decreases, respectively. Finally, five differentially expressed hsa-miRNAs were characterized by the highest expression in the adenoma samples and showed slightly decreased expression in the cancer group (see Table S3C).

### Microbial sRNAs in human stool samples.

All the reads previously not aligned to hsa-miRNAs and human sRNAs were further mapped against the human genome to identify those derived from human RNAs. On average, 61.9% of the input reads did not align to any human annotation (see Table S2). A tiny fraction of the remaining reads (average proportion of mapped reads = 0.004%) were aligned against miRNAs derived from animals or plants which can be commonly found in the Western diet. Their representation did not differ among the healthy, adenoma, and CRC subjects (average proportions of mapped reads, 0.004%, 0.004%, and 0.005%, respectively); therefore, we did not proceed further with the analyses in this direction.

Considering annotations of bacteria, archaea, and viruses in NCBI, an average of 18.93% of nonmapped human reads were aligned to microbial genome sequences, with the highest prevalence of reads assigned to bacteria (on average, 99.96% of the assigned reads) followed by archaea (average, 0.03%) and viruses (average, 0.03%) (see Table S2). Thus, we focused our analysis on the reads assigned to bacteria genomes to define a bacterial sRNA (bsRNA)-based profile (Fig. 1C; see also Table S1A to C).

**FIG 2:**
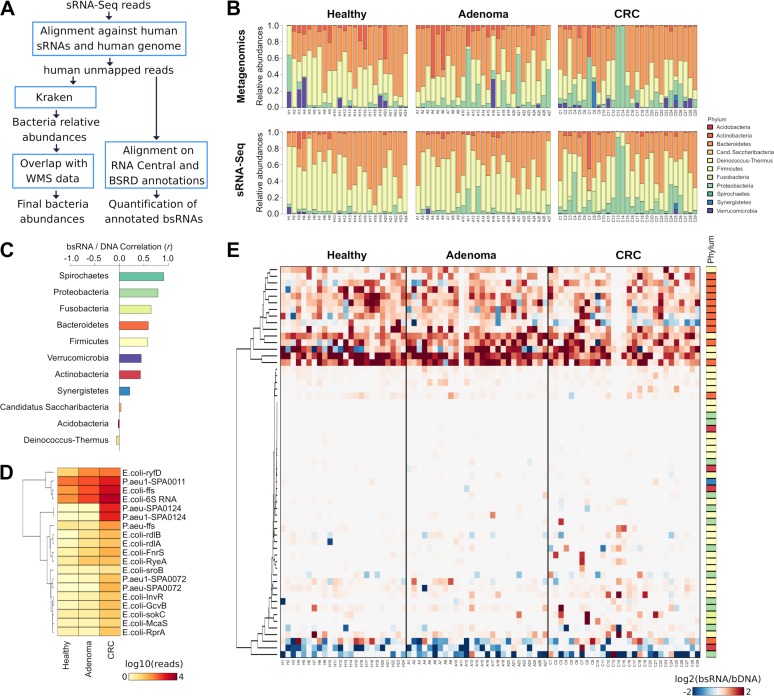
(A) Flow chart summarizing the analyses performed to identify and analyze the sRNA-Seq reads assigned to microbial genomes and bsRNAs. WMS, whole-metagenome sequencing. (B) Stacked bar plots reporting the relative abundances of bacterial phyla detected using whole-metagenome sequencing (top) and small RNA sequencing (sRNA-Seq; bottom) data, respectively. (C) Bar plot reporting the Pearson correlation coefficient (*r*) computed from comparisons between metagenomic and sRNA-Seq data for each phylum. (D) Heat map reporting the log2 ratios of relative abundances between bsRNAs and bacterial DNA profiles. Only ratios of bacterial species with median greater than 1 are reported. P.aeu, P. aeruginosa. (E) Heat map representing the log10 numbers of reads assigned to bsRNAs from the Bacteria Small RNA Database (BSRD). Only annotations that were significantly different (adjusted *P* value < 0.05) between healthy, adenoma, and CRC groups are shown.

The bsRNA reads were used to perform two types of analysis (Fig. 2A). The first analysis was based on the refinement of the bacterial profile obtained from the metagenomic data and the second on the identification of the differentially expressed bsRNAs. The information provided by the metagenomic experiments was integrated with that provided by the analysis of bsRNAs from sRNA-Seq data to improve the profiling of the bacteria. The consistency of the bsRNA-based profiles was verified by comparing the relative abundances of bacteria at each taxonomic level obtained from the metagenomic analysis (Fig. 2B) (see also Fig. S2A and B [https://doi.org/10.6084/m9.figshare.8080997] and Table S4A and B [https://doi.org/10.6084/m9.figshare.8080988] and Materials and Methods for more details). Considering all taxonomic levels, 50.2% of the annotations (*n* = 130) detected from DNA and RNA were significantly correlated (adjusted *P* value, <0.05) and all of them were associated with a positive correlation (median *r *=* *0.64) (see Table S4A).

At the species level, Porphyromonas asaccharolytica was characterized by the highest correlation (*r *=* *0.999; adjusted *P* value, <0.0001), while F. nucleatum (*r *=* *0.990; adjusted *P* value, <0.0001) and E. coli (*r *=* *0.632; adjusted *P* value, <0.0001) were among the 15 most highly correlated species (see Table S4C).

Considering the relative abundances from the bsRNA profiles, 15 species were significantly altered in the CRC group compared to the healthy group (see Table S1B). We confirmed that E. coli bsRNAs were more highly represented in the CRC than in the healthy group (adjusted *P* value, <0.0001) or the adenoma group (adjusted *P* value, <0.005), while levels of Bacteroides ovatus bsRNAs decreased in both the CRC group (adjusted *P* value, <0.05) and the adenoma group (adjusted *P* value, <0.05) compared to the healthy individuals.

The analysis of the content of local secondary structures in the reads of our sRNA-Seq data set showed that the reads assigned to bacteria had an overall lower minimum free energy (MFE) level than those assigned to humans (*P* value, <0.0001) (see Table S5A and B [https://doi.org/10.6084/m9.figshare.8080976]). That outcome is consistent with previous reports and observations of an increase in local secondary structures in bacterial RNAs (24). To further evaluate the nature of the bsRNAs detected by us, we quantified 21,088 nonredundant bsRNA annotations from RNA Central v12 (RNA Central Consortium 2018) (see Table S6A [https://doi.org/10.6084/m9.figshare.8080985]). Among them, 88.50% (*n* = 18,664) were annotated in RNA Central to only one bacterial species, particularly to E. coli (29.06% of the annotations). On average, 34.61% of the reads not mapping to the human genome in our sRNA-Seq data were assigned to these 18,664 annotations. tRNAs were the most highly represented biotype in our data (on average, 76.22% of read assignments), followed by rRNAs (on average, 23.72% of read assignments), and signal recognition particle (SRP) RNAs (on average, 0.02% of read assignments) (see Table S2). No differences were observed among the CRC, adenoma, and healthy control groups, whether or not the biotype annotation was considered (Wilcoxon rank sum test value, >0.05).

We identified 450 differentially expressed bsRNAs (adjusted *P* value < 0.05, median reads > 20) among the groups, and the highest number of bsRNAs (*n* = 419) was detected in comparison of the healthy and carcinoma groups (see Table S6B). The majority (*n* = 176, 42.0%) of these sRNAs were annotated to E. coli and were increasingly expressed in healthy, adenoma, and CRC subjects. This result is consistent with the higher abundance of E. coli in stool of CRC subjects as confirmed by the metagenomic data. Of note, other bacterial species were associated with differentially abundant sRNAs, including Eubacterium rectale, whose bsRNAs were less abundant in the CRC subjects, and Klebsiella pneumoniae, which was characterized by 52 bsRNAs that were more abundant in the CRC group. However, the results should be carefully evaluated because well-studied bacteria (particularly E. coli) are better annotated in these databases and because of the high level of sequence similarity existing between these annotations, particularly for tRNAs.

At this stage, we have verified whether other species-specific bsRNAs were characterized by dysregulated expression levels between sample groups. To reach this scope, we quantified the expression levels of bsRNAs annotated in the Bacteria Small RNA Database (BSRD) (25) and performed differential expression analyses among the groups. We identified 18 bsRNAs that were differentially expressed between the CRC and healthy control groups (Fig. 2D; see also Table S6C).

To complete the bsRNA analysis, we computed the relative abundances (ratios) of DNA and bsRNA (Fig. 2E; see also Table S4D). Fourteen species, mainly from the *Bacteroidetes* phylum, were characterized by a high transcription rate (high bsRNA/DNA ratio). Conversely, E. coli, Bifidobacterium longum, and Alistipes shahii showed low transcriptional rates (low bsRNA/DNA ratio).

### Specific signaling pathways targeted by hsa-miRNAs are correlated with Escherichia coli abundance.

**FIG 3:**
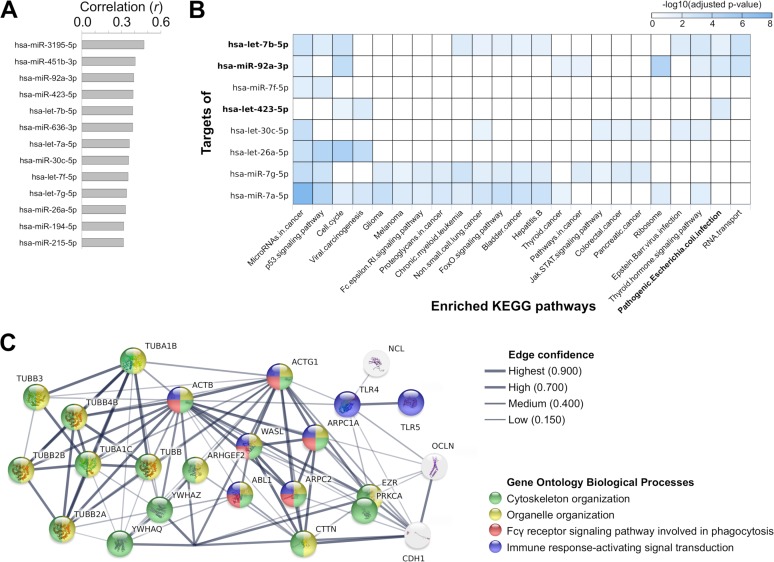
(A) Bar plot reporting the list of hsa-miRNAs and hsa-sncRNAs correlated with the abundance of E. coli. (B) Heat map showing the KEGG pathways significantly enriched in the targets of hsa-miRNAs correlated with E. coli abundance. Highlighted in bold are the hsa-miRNAs annotated as corresponding to the KEGG term “*Pathogenic* Escherichia coli *Infection*.” (C) STRING network representation of the interactions between genes belonging to the KEGG pathway “*Pathogenic* Escherichia coli *infection*” and targeted by hsa-miRNAs correlated with E. coli abundance. Nodes are colored based on their association with specific gene ontology biological processes. Edge thickness is proportional to the interaction confidence computed by STRING.

Given the observed increased abundance of E. coli DNA and bsRNA going from the healthy to the CRC group, we investigated a possible functional relationship with the altered hsa-miRNAs. As previously reported by Yuan and colleagues, a correlation between hsa-miRNA expression levels and bacterial abundances can be used to highlight candidate functional interactions (12). According to the results from the correlation analyses between the differentially expressed human sRNAs and the E. coli abundances, 13 hsa-miRNAs were significantly correlated with the E. coli abundances estimated using the metagenomic data (adjusted *P* value < 0.05) (Fig. 3A; see also Table S7A [https://doi.org/10.6084/m9.figshare.8080982]), with a trend of increased expression moving from the healthy to the carcinoma condition. No such correlation was seen when considering the hsa-sncRNAs other than the hsa-miRNAs.

To investigate the role of the 13 identified hsa-miRNAs in more detail, we performed a functional enrichment analysis of the human genes targeted by those hsa-miRNAs using miRPathDB (26). As expected, the most highly represented KEGG term was “*microRNA in cancer*,” which was enriched in the targets of six differentially expressed hsa-miRNAs (Fig. 3B). Interestingly, three of them (hsa-miR-423-5p, hsa-miR-92a-3p, and hsa-let-7b-5p) were also annotated to the KEGG term “*Pathogenic* Escherichia coli *Infection*.” To characterize the functional roles of the genes validated as targets of the three hsa-miRNAs referenced above, we performed an enrichment analysis using STRING (27) (see Table S7B). The biological processes significantly enriched for the 24 target genes were related to the KEGG terms “*cytoskeleton and organelle organization*,” “*Fc-gamma receptor signaling pathways*,” and “*immune-response*” (adjusted *P* value < 0.05) (Fig. 3C; see also Table S7C).

Using the WikiPathways annotations, the terms “*miRNA Regulation of DNA Damage Response*” and “*miRNAs involved in DNA damage response*” also emerged as statistically enriched in four other differentially expressed hsa-miRNAs (i.e., hsa-let-7a-5p, hsa-let-7b-5p, hsa-miR-26a-5p, and hsa-miR-7g-5p) correlated with the E. coli abundance (see Fig. S3A [https://doi.org/10.6084/m9.figshare.8080994] and Table S7B). As expected, the genes targeted by these four altered hsa-miRNAs are involved in the regulation of the response to radiation, signal transduction in response to DNA damage, and the mitotic cell cycle (see Fig. S3B and Table S7D).

### The combined use of human and microbial sRNAs improves classification.

**FIG 4:**
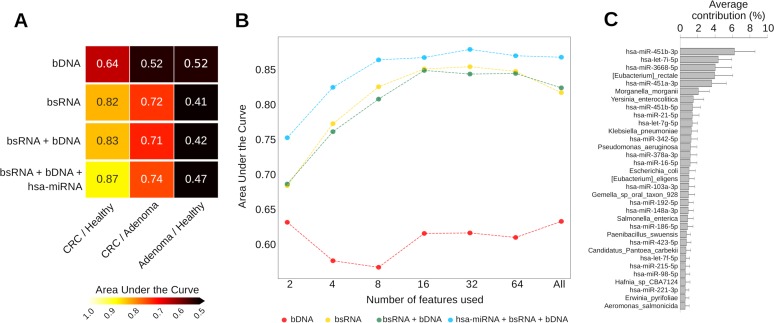
(A) Heat map reporting the area under the curve (AUC) computed by the Random Forest classifier using bacterial relative abundances provided by metagenomic data (bDNA), sRNA-Seq data (bsRNAs), and the combination of both bDNA and bsRNAs and combined with the expression levels of hsa-miRNAs (hsa-miRNAs + bDNA + bsRNAs). (B) Line plot reporting the AUC computed by the Random Forest classifier. For the classification of carcinoma and healthy samples, a specific number of features from different input information is reported. Rankings are obtained excluding testing set to avoid overfitting issues. (C) Bar plot reporting the average classification contribution of each of the 32 features providing the best classification accuracy of cancer and healthy samples. All the reported bacterial features were obtained using sRNA-Seq.

Finally, we tested a combination of transcriptomic and genomic profiles to classify the recruited subjects according to disease status. The Random Forest classification approach (4) provided high accuracy in classifying CRC cases and controls in unbiased cross-validation (area under the curve [AUC] = 0.86) in considering the profiles of both human and bacterial sRNA (hsa-miRNAs + bsRNAs) (see Fig. S4A and B [https://doi.org/10.6084/m9.figshare.8081006]). Conversely, the use of microbial DNA profiles alone showed lower potential with respect to classifying samples (AUC = 0.65). Note that the use of hsa-miRNA, bsRNA, and microbial DNA profiles provided the best classification performance for CRC versus controls (AUC = 0.87) and CRC versus adenomas (AUC = 0.74) (Fig. 4A). However, this signature was not able to distinguish between adenoma and controls samples (AUC = ∼0.5).

A signature of 32 features that included both hsa-miRNAs and bsRNA profiles from sRNA-Seq data provided the best differentiation of CRC subjects from healthy controls (Fig. 4B and C). The signature was composed of 57.7% human miRNAs and 42.4% microbial signals (Fig. 4C).

## DISCUSSION

The reproducibility of CRC-associated gut microbiome signatures among different populations may allow the development of new accurate oncobiome-based diagnostic models, as recently demonstrated by us (5, 6). However, a more accurate patient stratification may be obtained, considering not only the microbiome composition based on DNA but also the other biological molecules that can be retrieved in stool samples. In this study, we investigated the composition of human and microbial sRNAs in fecal samples from healthy subjects and patients with colorectal adenoma or carcinoma and we compared the different profiles among these groups. We showed that bsRNA profiles reflect the differences in the microbial DNA profiles characterizing each group. Moreover, our study demonstrated that a specific signature composed of profiles of human miRNAs, microbial sRNAs, and microbial DNAs was able to accurately classify the three groups of subjects with a high level of performance (AUC = 0.87), evidence that would be worth confirming across multiple cohorts.

In our analysis, the *Firmicutes* abundances characterizing cancer samples were significantly different from those characterizing either the healthy or the adenoma group. Interestingly, the *Verrucomicrobia* phylum, characterized by a significant peak of expression in the adenoma samples, may represent a potential candidate biomarker for precancer lesions. A significant increase in the level of the *Fusobacteria* phylum was also observed in the carcinoma group compared to the healthy and adenoma groups, as previously reported (5–7, 28–30). The abundance of F. nucleatum, a well-known CRC-related bacterium, increased from the healthy to the CRC group, albeit the variation observed was not statistically significant. Conversely, a significant increase in the abundance of E. coli was observed at both the DNA and bsRNA levels in our analysis, consistent with previous studies reporting an increase in the abundance of E. coli in the gut of CRC subjects (31–33). Furthermore, in the multicohort analysis performed by Thomas et al. (5), E. coli was the second-ranked bacterial species not only in our cohort (named Cohort1 in the paper) but also in another cohort from the United States which included metagenomic data of stool samples from 52 CRC patients and 52 healthy controls (34).

In recent years, researchers have focused on profiling microbial DNA or RNA in stool samples, marginally considering the microbial small RNA counterpart. In this respect, we have analyzed the nonmapped human reads from small RNA-Seq of stool samples in relation to metagenomic profiling. We observed that the microbial transcriptomic profiling was highly concordant with the metagenomic data, supporting the idea of combined use of the two deep-sequencing techniques. However, the current lack of an exhaustive catalog of sRNA annotations in nonmodel organisms forced us to restrict our microbial sRNA quantification only to a subset of well-known microbes. In fact, the majority of the identified bsRNAs differentially expressed between the subject groups were annotated to E. coli and Pseudomonas aeruginosa, which have been extensively studied in the field of molecular microbiology. Differently from E. coli, the relative abundance of P. aeruginosa in the CRC group was very low at both the DNA level (average abundance = 0.86) and the sRNA level (average abundance = 0.21), suggesting an inconsistent presence of sRNAs of this bacterium in our samples.

Our results showed that many E. coli bsRNAs annotated from both the RNA Central and BSRD databases increased in abundance going from healthy subjects to CRC patients. By the analysis of BSRD annotations, the 6S RNA coded by the *ssrS* gene of E. coli was the most abundant and significantly differentially expressed bsRNA. 6S RNA is a well-known transcriptional inhibitor that plays a pivotal role in the regulation of the stationary phase of bacteria and, under conditions of nutritional limitation, promotes bacterial survival (35). Other E. coli bsRNAs that were more abundant in the CRC group were *ryfD*, *ffs*, and *FnrS.* The overexpression of *ryfD* was associated with a decrease in the formation of E. coli biofilm and swarming ability (36). The *ffs* gene encodes 4.5S RNA, a part of the signal recognition particle involved in protein anchorage to the plasma membrane (37). Finally, the *FnrS* gene encodes a small regulatory RNA which regulates the cell metabolism in response to anaerobic growth conditions (38). A recent comparison of E. coli strains from colonic adenomas, adjacent colonic tissues, and normal mucosa revealed that the adenoma microenvironment promotes the selection of less motile and more pathogenic strains (33). An increase of specific E. coli bsRNAs in stool samples from patients with CRC or adenoma, as observed in our study, could reflect the progressive selection of specific strains colonizing the tumor microenvironment.

Interestingly, a subset of genes targeted by upregulated hsa-miRNAs in CRC subjects codes for proteins interacting with specific E. coli proteins. Most of these proteins are involved in the tubulin/actin-based cytoskeleton organization which is extensively altered by the EspG and EspG2 proteins produced by enteropathogenic E. coli infection (39). Among other enriched functional terms was “*Fc-gamma receptor signaling pathway involved in phagocytosis*,” a pathway involving bacterial phagocytosis by immune cells as well as enterocytes (40, 41). Furthermore, two Toll-like receptor-coding genes (*TLR5* and *TLR4*) were also targeted by the hsa-miRNAs identified in our study. TLR4 is involved in the innate immune response to bacterium recognition (42), but it is also necessary and sufficient for bacterium phagocytosis by IEC-6 intestinal epithelial cells (42, 43).

Evidence of a role of hsa-miRNAs in the modulation of the immune response to specific pathogens was reported previously (10), and an altered form of hsa-miRNA expression that was detected in *in vivo* and *in vitro* models of E. coli infection was also reported previously (44–46). For example, hsa-miRNA-30c was previously identified as upregulated in T84 cells and mouse enterocytes during adherent-invasive E. coli infection (46); on the other hand, let-7b overexpression reduced the severity of colitis induced by adherent-invasive E. coli infection in mouse models of Crohn’s disease (47). More experiments are required to further characterize the specific response to E. coli during CRC progression since the induction of various hsa-miRNAs or their homologs in mice was observed in response to different pathogens and lipopolysaccharide molecules (48, 49). Despite the limitations of correlation analysis and the requirement for more *in vitro* validation experiments, our data support the hypothesis of an interaction between hsa-miRNAs and bacteria via target genes involved in the bacterium adhesion and phagocytosis pathways.

Another functional annotation enriched in the gene targets of hsa-miRNAs correlated with E. coli abundance was the DNA damage response. The enrichment of genes involved in this pathway could be relevant given the evidence of E. coli colonization in microsatellite unstable CRC (50) and of the genotoxic effect of the colibactin protein produced by *pks*-positive (*pks*^+^) E. coli strains which were enriched in stool samples and biopsy specimens from CRC subjects (51, 52). However, we observed only a nonsignificantly higher abundance of *pks*^+^ E. coli in the CRC group than in the healthy subjects (data not shown), suggesting the prevalence of other strains in our samples.

The differential expression analysis suggested hsa-miR-30-5p as a candidate biomarker for adenomas given its peak of expression in this group. Similarly, the five hsa-miRNAs significantly upregulated in the CRC group compared to the adenoma and healthy groups (hsa-miR-21-5p, hsa-miR-200b-3p, hsa-miR-1290-5p, hsa-miR-4792-3p, and hsa-miR-1246-3p) could be considered promising CRC biomarkers.

Another critical aspect to consider is whether stool data reflect the sRNA expression profile and microbiome composition differences between the CRC primary tissue and the adjacent normal colonic mucosa. We compared the 19 hsa-miRNAs belonging to our signature defined by the Random Forest classifier with seven publicly available data sets of hsa-miRNAs differentially expressed between CRC tissues and matched adjacent colonic mucosa (see Table S8A [https://doi.org/10.6084/m9.figshare.8080979]). All the overlaps were statistically significant and involved from 4 to 12 hsa-miRNAs of our signature. Hsa-miR-21-5p was the only example from our signature detected as differentially expressed in all the compared data sets, followed by hsa-miR-378a-3p (differentially expressed in six data sets) and hsa-miR-215-5p (differentially expressed in five data sets) (see Table S8B). Considering also public annotations of hsa-miRNAs dysregulated in CRC primary tissues (miRCancer database), the hairpin sequences of 13 mature hsa-miRNAs belonging to the Random Forest signature were detected as annotated to CRC disease in this database (see Table S8C).

Yuan and colleagues performed both miRNA profiling and 16S RNA profiling of 44 CRC and paired healthy tissues (12). The comparison of these data with the hsa-miRNAs of our signature shows an overlap of four hsa-miRNAs (hsa-miR-21-5p, hsa-miR-148a-3p, hsa-miR-378a-3p, and hsa-miR-98-5p) detected as dysregulated in both studies (see Table S8A and B). However, in contrast to the study of Yuan and colleagues, we found a significant increase in E. coli abundance in stool samples from CRC subjects whereas F. nucleatum abundance was increased, albeit not significantly. This result is consistent with the heterogeneity of microbial profiles that we observed in recent multicohort analyses of metagenomic data in stool samples from CRC subjects (5, 6).

The results presented here took advantage of the use of dual pools of information provided by the concomitant DNA and sRNA sequencing of the same stool samples. This experimental setting provided us an overview of the microbial population and their activities. Considering the CRC microbiome profiles annotated in the Disbiome database (53), we obtained evidence of a relation with CRC for six species characterized by high metagenomic/sRNA-Seq correlation (P. asaccharolytica, F. nucleatum, B. fragilis, E. coli, Enterococcus faecalis, and Alistipes finegoldii) (see Table S4C and Table S9 [https://doi.org/10.6084/m9.figshare.8342552]). P. asaccharolytica was the bacterium characterized by the highest correlation, and it was found in abundance in stool or tissue biopsy specimens of CRC subjects in six studies, including the multicohort analyses performed by Thomas et al. and Wirbel et al. (5, 6). Similarly, F. nucleatum and B. fragilis were detected as abundant in stool or tissue biopsy specimens from CRC subjects in different studies, and they were characterized by a high DNA/bsRNA correlation in our analysis. These results suggested that the bacterial species associated with CRC lesions are transcriptionally active and release small RNAs whose role in CRC could be relevant.

Although microbial genomic sequences are becoming exhaustively available, thanks to the diffusion of metagenomic analyses, a comprehensive database of microbial annotations is still needed at the RNA level. In this respect, we aligned the sRNA-Seq reads first to microbial genomes and, subsequently, to RNA Central annotations or BSRD annotations separately. It is important to highlight that only specific microbial species, particularly strains of E. coli, are stored in both databases. Different computational strategies have been proposed to predict novel microbial sRNAs based on their primary sequence and secondary structure. However, to improve the catalog of annotated bsRNAs (54, 55), larger cohorts are needed to provide proper sRNA predictions based on sRNA-Seq reads, and this is beyond the scope of the present study.

From the clinical point of view, our analysis provided further support for the idea of the use of a combination of fecal microbial and human RNA biomarkers to better distinguish subjects with colonic adenoma or carcinoma from healthy individuals. The isolation of RNA from stool samples can provide combinatorial information on the small RNA expression profile of the host and the gut microbiome that can be used for accurate classification of the subjects according to their health status. Validation on a larger independent cohort of patients and healthy controls is mandatory to assess the accuracy of these biomarkers. However, to the best of our knowledge, at the moment, there is no similar study/cohort available that collected RNA and DNA from the same stool samples and performed concomitant sequencing analyses. Our integrative analyses provided further evidence of an altered host-gut microbiome relationship in cancer and suggested an unexplored role of bsRNAs in CRC. Furthermore, the characterization of gut microbiome sRNA molecules could provide a novel opportunity to improve CRC treatments in light of the role of microbiota in the modulation of the patient response to cancer chemotherapy and immunotherapy (56, 57).

## MATERIALS AND METHODS

### Study population.

Samples were collected from patients recruited in a hospital-based study at the Clinica S. Rita in Vercelli, Italy. On the basis of colonoscopy results, participants were classified into three categories: (i) healthy subjects (individuals with colonoscopy results negative for tumor, adenomas, and inflammatory bowel disease [IBD]); (ii) adenoma patients (individuals with a colorectal adenoma[s]); and (iii) colorectal cancer patients (individuals with newly diagnosed CRC). A total of 80 subjects (29 CRC patients, 27 adenoma, and 24 controls) were included in the present study. CRC patients were recruited at the first CRC diagnosis and had not received any treatment before the fecal sample collection.

The study was approved by the local ethics committee (ethics committee of Azienda Ospedaliera SS. Antonio e Biagio e C. Arrigo of Alessandria, Italy; protocol N. Colorectal miRNA CEC2014), and informed consent was obtained from all participants.

### Sample collection.

Naturally evacuated fecal samples were obtained from all patients previously instructed to self-collect the specimen at home before any bowel preparation for colonoscopy. The stool was collected in stool nucleic acid collection and transport tubes with RNA stabilizing solution (Norgen Biotek Corp.) and returned at the time of performing a colonoscopy in the endoscopy unit or at the time of blood sampling. Aliquots (200 ml) of the stool samples were stored at –80°C until RNA/DNA extraction.

### Extraction of total RNA and total DNA from stool.

RNA was extracted using a stool total RNA purification kit (Norgen Biotek Corp.). The RNA quality and quantity were verified according to the MIQE guidelines (http://miqe.gene-quantification.info/). The RNA concentration was quantified by Qubit with a Qubit microRNA assay kit (Invitrogen). The DNA extraction was performed with a QIAamp DNA stool minikit (Qiagen, Hilden, Germany) according to the instructions of the manufacturer. Finally, DNA was eluted in 100 μl of the elution buffer provided with the kit. The DNA quantification was performed with a Qubit DNA high-sensitivity (HS) assay kit (Invitrogen).

### Analysis of microbiome composition by shotgun sequencing.

Sequencing libraries were prepared using a Nextera XT DNA library preparation kit (Illumina, CA, USA), following the manufacturer’s guidelines and a previously reported protocol (22, 23). Sequencing was performed on a HiSeq 2500 sequencer (Illumina, CA, USA) at the internal sequencing facility of the Centre for Integrative Biology, Trento, Italy. Whole-metagenome sequencing data were analyzed as described previously (5). Analysis of differential abundances between subject groups was performed using the Wilcoxon rank sum test. Only data from species with average abundances higher than 0.1% in at least one group and with Benjamini-Hochberg (BH)-adjusted *P* values of <0.05 were considered statistically significant.

### Library preparation for small RNA sequencing (sRNA-Seq).

sRNA transcripts were converted into barcoded cDNA libraries. Library preparation was performed with a NEBNext multiplex small RNA library prep set for Illumina (protocol E7330; New England BioLabs Inc., USA) as described previously (22). For each sample, 250 ng of RNA was used as the starting material to prepare libraries. Each library was prepared with a unique indexed primer so that the libraries could all be pooled into one sequencing lane. Multiplex adapter ligations, reverse transcription primer hybridization, reverse transcription reactions, and the PCR amplification were performed as described in the protocol provided by the manufacturer. After PCR preamplification, the cDNA constructs were purified with a QIAQuick PCR purification kit (Qiagen, Germany) following the modifications suggested in the NEBNext multiplex small RNA library prep protocol. Further quality control checks and size selections were performed following the NEBNext multiplex small RNA library prep protocol (protocol E7330; New England BioLabs Inc., USA). Size selection of the amplified cDNA constructs was performed using Novex Tris-borate-EDTA (TBE) gels (Invitrogen) (6%) and following the procedure of gel electrophoresis running and purification of the construct described in the Illumina TruSeq small RNA library prep protocol. The 140-nt and 150-nt bands correspond to adapter-ligated constructs derived from RNA fragments of 21 to 30 nt. A concluding Bioanalyzer 2100 run performed with a high-sensitivity DNA kit (Agilent Technologies, Germany) permitted checking final size, purity, and concentration for the sequences in the DNA libraries.

The obtained libraries (24 samples were multiplexed) were subjected to the Illumina sequencing pipeline, passing through clonal cluster generation on a single-read flow cell (Illumina Inc., USA) by bridge amplification on a cBot (TruSeq SR cluster kit, v3-cBOT-HS; Illumina Inc., USA) and 50 cycles of sequencing by synthesis using a HiSeq 2000 sequencer (Illumina Inc., USA) (in collaboration with Genecore Facility at EMBL, Heidelberg, Germany).

### Analysis of sRNA from sRNA-seq data.

sRNA-Seq pipeline analyses were performed using a previously described approach (22, 23). Fastq files were quality checked (QC) using FastQC software (http://www.bioinformatics.babraham.ac.uk/projects/fastqc/). Reads shorter than 14 nt were discarded. The QC-passed reads were clipped from the adapter sequences using Cutadapt (58) by imposing a maximum error rate in terms of mismatches, insertions, and deletions equal to 0.15. The length of the raw sRNA-Seq reads was 50 bp, and the reads were, on average, 24.8 bp in length after adapter removal. Trimmed reads were mapped against an in-house reference of human sRNA sequences composed of (i) 1,881 precursor hsa-miRNAs from miRBase v21 (59); (ii) 32,826 hsa-piRNA sequences from piRBase v1.0 (60); and (iii) 5,171 hsa-sncRNA sequences shorter than 80 bp from Database of Small Human noncoding RNAs (DASHR) database v1.0 (61). The alignment was performed using BWA algorithm v. 0.7.12 (62) with the default settings. Using these settings, the seeding was not performed for reads shorter than 32 bp, and the reads were entirely evaluated for the alignment. The hsa-miRNAs were annotated and quantified using two methods called the “knowledge-based” and “position-based” methods. In the “knowledge-based” method, the arm starting positions of the hsa-miRNAs precursor were well defined. On the basis of the position of the mapped reads, it was possible to quantify the mature hsa-miRNAs. Alternatively, the “position-based” method was implemented for those hsa-miRNA precursors in which the positions of the 5′ and 3′ arms are not defined. With the second method, on the basis of the position of the read, the name of hsa-miRNAs was assigned a “5′ Novel” or “3′ Novel” suffix and the data were quantified. The results generated by the annotation and quantification methods were merged into a unique mature hsa-miRNA count matrix.

The quantification of hsa-sncRNAs annotations was performed by counting the read alignment reported by BWA output sam files. The reads that were left unmapped in the annotations of the hsa-miRNAs and hsa-sncRNAs were aligned against the human hg38 genome using BWA with the default settings. The reads that were left unmapped on the human genome were then aligned against annotations of bacterial genomes and sRNAs.

Differential expression analysis was performed with DESeq2 R package v.1.22.2 (63) using the likelihood ratio test (LRT) function. This function was selected in order to correct the analysis, including age and gender as covariates. A gene was defined as differentially expressed if associated with a BH-adjusted *P* value of less than 0.05 and supported by at least a median number of reads higher than 20 within at least one of the sample groups considered.

Reads that were left unmapped to the human genome were considered for the quantification of annotations of nonhuman sRNAs. Using these data, quantification of diet-related miRNAs was performed by considering 5,293 miRNAs annotated to animal or plant species which are part of a Western diet and may be retrievable in human stool. The annotation of cancer-related hsa-miRNAs retrieved from the miRCancer database (64) was performed using the version from 18 February 2019. Only annotations obtained from studies performed on CRC tissues were considered.

### Estimation of microbial sRNA expression levels.

The sRNA-Seq reads that were not aligned were mapped against microbial genomes using the Kraken algorithm (65). Kraken was applied in the default setting by considering bacterial, archaeal, and viral genomes from NCBI. Using these settings, only reads with length ≥31 bp were used for the analysis (average, 30.71% of human unmapped reads). The fraction of reads analyzed by Kraken and the average read length in each sample are reported in Table S2. The Kraken output in MetaPhlAn2 format obtained with the option –mpa-format was used in the analysis. For each taxonomic level, the number of reads assigned to microbial annotations by Kraken was converted into relative abundances using the total number of assigned reads.

Quantification of bsRNAs annotated in RNA Central v12 (66) was performed by considering only bacterial species characterized by an average abundance higher than 0.01% in our metagenomic data. Considering these bacterial species, 286,433 bsRNAs with a length of <80 bp were retrieved from the database and were merged into a set of 21,088 nonredundant sequences (since identical sequences were annotated to different species or strains) (see Table S6A). The expression level of BSRD annotations (25) was quantified using BWA by aligning human-unmapped reads against their sequence.

Differential expression analysis of RNA Central and BSRD annotations was performed using the DESeq2 R package (63).

### Comparison and integration of MetaPhlAn2 and Kraken results.

The comparison between MetaPhlAn2 and Kraken relative abundances was performed by considering separately the abundances computed at each taxonomic level. Correlation analysis was performed using the Pearson method and the R function “cor” and correlation *P* value computed using the “cor.test” R function. Multiple-test correction was performed using the BH method implemented in the “p.adjust” R function. Only correlations associated with a BH-adjusted *P* value lower than 0.05 were considered significant. The most highly correlated species were identified by considering only the species with an average abundance greater than 0.1% in at least one sample group, a Pearson *r* higher than 0.6, and a BH-adjusted *P* value lower than 0.05.

The estimation of bsRNA transcriptional rate was computed by dividing the bacterial relative abundance computed by Kraken by the abundances computed by MetaPhlAn2.

Analysis of bacterial species annotated to CRC disease was performed using the annotation from the Disbiome database (53).

### Secondary structure prediction.

Prediction of the secondary structure of reads annotated to bsRNAs and the associated MFE level were computed using the RNAFold algorithm (67). The algorithm was applied to reads assigned to hsa-miRNAs, non-miRNA sRNA annotations, the human genome, or bsRNAs or not aligned. For each data set, the average MFE was computed. The statistical significance of MFE differences among read types was computed using the Wilcoxon rank sum test.

### Analysis of hsa-miRNAs correlated with Escherichia coli.

Correlations between E. coli abundance (defined by the metagenomic data) and expression levels of hsa-miRNAs and hsa-sncRNAs were computed using the Pearson method. Only genes associated with a correlation BH-adjusted *P* value of <0.05 were considered. The set of functional processes enriched in the targets of E. coli-correlated hsa-miRNAs was defined using miRPathDB tool v1.1 (26). Only the processes significantly enriched (BH-adjusted *P* value < 0.05) for at least two hsa-miRNAs were selected. The functional terms annotated in the KEGG and WikiPathway databases were considered for the analysis.

The list of Gene Ontology Biological Processes enriched for the list of genes targeted by E. coli-correlated hsa-miRNAs and annotated to the annotations *Pathogenic* Escherichia coli *Infection* or *miRNA Regulation of DNA Damage Response* was defined using the analysis module of the STRING v11.0 tool (27). The top 20 annotations associated with a BH-adjusted *P* value of <0.05 were selected.

### Machine learning approach for sample classification.

A Random Forest classifier was applied to classify each class of subjects, as described previously (5). Specifically, four types of the obtained quantitative profiles, namely, bDNA, bsRNA, hsa-miRNA, and hsa-sncRNA, were considered, together with any combination of the four, for a total of 14 different data categories. For each type of data, three different classification comparisons were considered: CRC versus healthy, CRC versus adenoma, and adenoma versus healthy. The CRC class, when present, has been considered the positive class; the adenoma class has been considered the positive class in the lattermost combination. The total number of machine learning experiments was 42. Each experiment consisted of a 10-fold cross-validation iterated 20 times. Folds contained comparable numbers of positive-class and negative-class representatives. The AUC was computed as the average among 200 tests for each comparison. Feature rankings from the Random Forest shown in this work have been rigorously obtained by considering each time the sole training set to avoid overestimations due to overfitting issues.

### Evaluation of the identified stool hsa-miRNA signature in CRC tissues from public datasets.

The expression of hsa-miRNAs belonging to the feature set providing the best classification of CRC and healthy subjects was evaluated in independent analyses of hsa-miRNA expression in primary CRC tissues and adjacent colonic mucosa. In detail, the expression of 19 hsa-miRNAs belonging to the Random Forest signature was compared with that seen with three data sets of differentially expressed hsa-miRNAs (“Mjelle et al., 2019” [69], “Neerincx et al., 2015” [70], and “Sun et al., 2016” [71] sets) from Table S2 in reference 15, the set of 76 differentially expressed hsa-miRNAs from Table S1 in reference 12, and the set of hsa-miRNAs differentially expressed between CRC and adjacent tissues as obtained from the reanalysis of three microarray experiments (GEO accession no. GSE108153, GSE115513, and GSE35834). Differential expression analysis of microarray data was performed using the GEO2R tool, and microarray probes were converted to miRBase v21 annotations using miRiadne (68). For each analysis, only significantly differentially expressed hsa-miRNAs were considered (adjusted *P* value < 0.05) and the overlap of the numbers of these hsa-miRNAs and those from the Random Forest signature was statistically evaluated using Fisher’s exact test.

### Data availability.

Raw sRNA-Seq data are deposited in Gene Expression Omnibus (GEO) with the identifier GSE132236. Raw metagenomic data are deposited in the Sequence Read Archive (SRA) database with the identifier SRP136711.

## References

[1] Yu J, Feng Q, Wong SH, Zhang D, Liang QY, Qin Y, Tang L, Zhao H, Stenvang J, Li Y, Wang X, Xu X, Chen N, Wu WKK, Al-Aama J, Nielsen HJ, Kiilerich P, Jensen BAH, Yau TO, Lan Z, Jia H, Li J, Xiao L, Lam TYT, Ng SC, Cheng A-L, Wong V-S, Chan FKL, Xu X, Yang H, Madsen L, Datz C, Tilg H, Wang J, Brünner N, Kristiansen K, Arumugam M, Sung JJ-Y, Wang J 2017 Metagenomic analysis of faecal microbiome as a tool towards targeted non-invasive biomarkers for colorectal cancer. Gut 66:70–78. doi:10.1136/gutjnl-2015-309800. 26408641

[2] Tilg H, Adolph TE, Gerner RR, Moschen AR 2018 The intestinal microbiota in colorectal cancer. Cancer Cell 33:954–964. doi:10.1016/j.ccell.2018.03.004. 29657127

[3] Garrett WS 2015 Cancer and the microbiota. Science 348:80–86. doi:10.1126/science.aaa4972. 25838377 PMC5535753

[4] Brennan CA, Garrett WS 2016 Gut microbiota, inflammation, and colorectal cancer. Annu Rev Microbiol 70:395–411. doi:10.1146/annurev-micro-102215-095513. 27607555 PMC5541233

[5] Thomas AM, Manghi P, Asnicar F, Pasolli E, Armanini F, Zolfo M, Beghini F, Manara S, Karcher N, Pozzi C, Gandini S, Serrano D, Tarallo S, Francavilla A, Gallo G, Trompetto M, Ferrero G, Mizutani S, Shiroma H, Shiba S, Shibata T, Yachida S, Yamada T, Wirbel J, Schrotz-King P, Ulrich CM, Brenner H, Arumugam M, Bork P, Zeller G, Cordero F, Dias-Neto E, Setubal JC, Tett A, Pardini B, Rescigno M, Waldron L, Naccarati A, Segata N 2019 Metagenomic analysis of colorectal cancer datasets identifies cross-cohort microbial diagnostic signatures and a link with choline degradation. Nat Med 25:667–678. doi:10.1038/s41591-019-0405-7. 30936548 PMC9533319

[6] Wirbel J, Pyl PT, Kartal E, Zych K, Kashani A, Milanese A, Fleck JS, Voigt AY, Palleja A, Ponnudurai R, Sunagawa S, Coelho LP, Schrotz-King P, Vogtmann E, Habermann N, Niméus E, Thomas AM, Manghi P, Gandini S, Serrano D, Mizutani S, Shiroma H, Shiba S, Shibata T, Yachida S, Yamada T, Waldron L, Naccarati A, Segata N, Sinha R, Ulrich CM, Brenner H, Arumugam M, Bork P, Zeller G 2019 Meta-analysis of fecal metagenomes reveals global microbial signatures that are specific for colorectal cancer. Nat Med 25:679–689. doi:10.1038/s41591-019-0406-6. 30936547 PMC7984229

[7] Feng Q, Liang S, Jia H, Stadlmayr A, Tang L, Lan Z, Zhang D, Xia H, Xu X, Jie Z, Su L, Li X, Li X, Li J, Xiao L, Huber-Schönauer U, Niederseer D, Xu X, Al-Aama JY, Yang H, Wang J, Kristiansen K, Arumugam M, Tilg H, Datz C, Wang J 2015 Gut microbiome development along the colorectal adenoma-carcinoma sequence. Nat Commun 6:6528. doi:10.1038/ncomms7528. 25758642

[8] Williams MR, Stedtfeld RD, Tiedje JM, Hashsham SA 2017 MicroRNAs-based inter-domain communication between the host and members of the gut microbiome. Front Microbiol 8:1896. doi:10.3389/fmicb.2017.01896. 29021788 PMC5624305

[9] Liu S, da Cunha AP, Rezende RM, Cialic R, Wei Z, Bry L, Comstock LE, Gandhi R, Weiner HL 2016 The host shapes the gut microbiota via fecal microRNA. Cell Host Microbe 19:32–43. doi:10.1016/j.chom.2015.12.005. 26764595 PMC4847146

[10] Zhou X, Li X, Wu M 2018 miRNAs reshape immunity and inflammatory responses in bacterial infection. Signal Transduct Target Ther 3:14. doi:10.1038/s41392-018-0006-9. 29844933 PMC5968033

[11] Dalmasso G, Nguyen HTT, Yan Y, Laroui H, Charania MA, Ayyadurai S, Sitaraman SV, Merlin D 2011 Microbiota modulate host gene expression via microRNAs. PLoS One 6:e19293. doi:10.1371/journal.pone.0019293. 21559394 PMC3084815

[12] Yuan C, Burns MB, Subramanian S, Blekhman R 2018 Interaction between host microRNAs and the gut microbiota in colorectal cancer. mSystems 3:e00205-17. doi:10.1128/mSystems.00205-17. 29795787 PMC5954203

[13] Proença MA, Biselli JM, Succi M, Severino FE, Berardinelli GN, Caetano A, Reis RM, Hughes DJ, Silva AE 2018 Relationship between Fusobacterium nucleatum, inflammatory mediators and microRNAs in colorectal carcinogenesis. World J Gastroenterol 24:5351–5365. doi:10.3748/wjg.v24.i47.5351. 30598580 PMC6305535

[14] Yang Y, Weng W, Peng J, Hong L, Yang L, Toiyama Y, Gao R, Liu M, Yin M, Pan C, Li H, Guo B, Zhu Q, Wei Q, Moyer M-P, Wang P, Cai S, Goel A, Qin H, Ma Y 2017 Fusobacterium nucleatum increases proliferation of colorectal cancer cells and tumor development in mice by activating Toll-like receptor 4 signaling to nuclear factor-κB, and up-regulating expression of microRNA-21. Gastroenterology 152:851–866.e24. doi:10.1053/j.gastro.2016.11.018. 27876571 PMC5555435

[15] Mjelle R, Sjursen W, Thommesen L, Sætrom P, Hofsli E 2019 Small RNA expression from viruses, bacteria and human miRNAs in colon cancer tissue and its association with microsatellite instability and tumor location. BMC Cancer 19:161. doi:10.1186/s12885-019-5330-0. 30786859 PMC6381638

[16] Dutcher HA, Raghavan R 2018 Origin, evolution, and loss of bacterial small RNAs. Microbiol Spectr 6(2). doi:10.1128/microbiolspec.RWR-0004-2017. PMC5890949 29623872

[17] Ahmed W, Hafeez MA, Mahmood S 2018 Identification and functional characterization of bacterial small non-coding RNAs and their target: a review. Gene Rep 10:167–176. doi:10.1016/j.genrep.2018.01.001.

[18] Nitzan M, Rehani R, Margalit H 2017 Integration of bacterial small RNAs in regulatory networks. Annu Rev Biophys 46:131–148. doi:10.1146/annurev-biophys-070816-034058. 28532217

[19] Strong MJ, Xu G, Morici L, Splinter Bon-Durant S, Baddoo M, Lin Z, Fewell C, Taylor CM, Flemington EK 2014 Microbial contamination in next generation sequencing: implications for sequence-based analysis of clinical samples. PLoS Pathog 10:e1004437. doi:10.1371/journal.ppat.1004437. 25412476 PMC4239086

[20] Mangul S, Yang HT, Strauli N, Gruhl F, Porath HT, Hsieh K, Chen L, Daley T, Christenson S, Wesolowska-Andersen A, Spreafico R, Rios C, Eng C, Smith AD, Hernandez RD, Ophoff RA, Santana JR, Levanon EY, Woodruff PG, Burchard E, Seibold MA, Shifman S, Eskin E, Zaitlen N 2018 ROP: dumpster diving in RNA-sequencing to find the source of 1 trillion reads across diverse adult human tissues. Genome Biol 19:36. doi:10.1186/s13059-018-1403-7. 29548336 PMC5857127

[21] Quince C, Walker AW, Simpson JT, Loman NJ, Segata N 2017 Shotgun metagenomics, from sampling to analysis. Nat Biotechnol 35:833–844. doi:10.1038/nbt.3935. 28898207

[22] Ferrero G, Cordero F, Tarallo S, Arigoni M, Riccardo F, Gallo G, Ronco G, Allasia M, Kulkarni N, Matullo G, Vineis P, Calogero RA, Pardini B, Naccarati A 2018 Small non-coding RNA profiling in human biofluids and surrogate tissues from healthy individuals: description of the diverse and most represented species. Oncotarget 9:3097–3111. doi:10.18632/oncotarget.23203. 29423032 PMC5790449

[23] Kulkarni N, Alessandrì L, Panero R, Arigoni M, Olivero M, Ferrero G, Cordero F, Beccuti M, Calogero RA 2018 Reproducible bioinformatics project: a community for reproducible bioinformatics analysis pipelines. BMC Bioinformatics 19:349. doi:10.1186/s12859-018-2296-x. 30367595 PMC6191970

[24] Katz L, Burge CB 2003 Widespread selection for local RNA secondary structure in coding regions of bacterial genes. Genome Res 13:2042–2051. doi:10.1101/gr.1257503. 12952875 PMC403678

[25] Li L, Huang D, Cheung MK, Nong W, Huang Q, Kwan HS 2013 BSRD: a repository for bacterial small regulatory RNA. Nucleic Acids Res 41:D233–D238. doi:10.1093/nar/gks1264. 23203879 PMC3531160

[26] Backes C, Kehl T, Stöckel D, Fehlmann T, Schneider L, Meese E, Lenhof H-P, Keller A 2017 miRPathDB: a new dictionary on microRNAs and target pathways. Nucleic Acids Res 45:D90–D96. doi:10.1093/nar/gkw926. 27742822 PMC5210630

[27] Szklarczyk D, Gable AL, Lyon D, Junge A, Wyder S, Huerta-Cepas J, Simonovic M, Doncheva NT, Morris JH, Bork P, Jensen LJ, von Mering C 2019 STRING v11: protein–protein association networks with increased coverage, supporting functional discovery in genome-wide experimental datasets. Nucleic Acids Res 47:D607–D613. doi:10.1093/nar/gky1131. 30476243 PMC6323986

[28] Santoru ML, Piras C, Murgia A, Palmas V, Camboni T, Liggi S, Ibba I, Lai MA, Orrù S, Blois S, Loizedda AL, Griffin JL, Usai P, Caboni P, Atzori L, Manzin A 2017 Cross sectional evaluation of the gut-microbiome metabolome axis in an Italian cohort of IBD patients. Sci Rep 7:9523. doi:10.1038/s41598-017-10034-5. 28842640 PMC5573342

[29] Zeller G, Tap J, Voigt AY, Sunagawa S, Kultima JR, Costea PI, Amiot A, Böhm J, Brunetti F, Habermann N, Hercog R, Koch M, Luciani A, Mende DR, Schneider MA, Schrotz-King P, Tournigand C, Tran Van Nhieu J, Yamada T, Zimmermann J, Benes V, Kloor M, Ulrich CM, von Knebel Doeberitz M, Sobhani I, Bork P 2014 Potential of fecal microbiota for early-stage detection of colorectal cancer. Mol Syst Biol 10:766. doi:10.15252/msb.20145645. 25432777 PMC4299606

[30] Dai Z, Coker OO, Nakatsu G, Wu WKK, Zhao L, Chen Z, Chan FKL, Kristiansen K, Sung JJY, Wong SH, Yu J 2018 Multi-cohort analysis of colorectal cancer metagenome identified altered bacteria across populations and universal bacterial markers. Microbiome 6:70. doi:10.1186/s40168-018-0451-2. 29642940 PMC5896039

[31] Wassenaar TM 2018 E. coli and colorectal cancer: a complex relationship that deserves a critical mindset. Crit Rev Microbiol 44:619–632. doi:10.1080/1040841X.2018.1481013. 29909724

[32] Sears CL, Garrett WS 2014 Microbes, microbiota, and colon cancer. Cell Host Microbe 15:317–328. doi:10.1016/j.chom.2014.02.007. 24629338 PMC4003880

[33] Ambrosi C, Sarshar M, Aprea MR, Pompilio A, Di Bonaventura G, Strati F, Pronio A, Nicoletti M, Zagaglia C, Palamara AT, Scribano D 2019 Colonic adenoma-associated Escherichia coli express specific phenotypes. Microbes Infect 2019:S1286-4579(19)30008-5. doi:10.1016/j.micinf.2019.02.001. 30763764

[34] Vogtmann E, Hua X, Zeller G, Sunagawa S, Voigt AY, Hercog R, Goedert JJ, Shi J, Bork P, Sinha R 2016 Colorectal cancer and the human gut microbiome: reproducibility with whole-genome shotgun sequencing. PLoS One 11:e0155362. doi:10.1371/journal.pone.0155362. 27171425 PMC4865240

[35] Cavanagh AT, Wassarman KM 2014 6S RNA, a global regulator of transcription in Escherichia coli, Bacillus subtilis, and beyond. Annu Rev Microbiol 68:45–60. doi:10.1146/annurev-micro-092611-150135. 24742053

[36] Bak G, Lee J, Suk S, Kim D, Young Lee J, Kim K-S, Choi B-S, Lee Y 2015 Identification of novel sRNAs involved in biofilm formation, motility, and fimbriae formation in Escherichia coli. Sci Rep 5:15287. doi:10.1038/srep15287. 26469694 PMC4606813

[37] Luirink J, Dobberstein B 1994 Mammalian and Escherichia coli signal recognition particles. Mol Microbiol 11:9–13. doi:10.1111/j.1365-2958.1994.tb00284.x. 8145649

[38] Boysen A, Møller-Jensen J, Kallipolitis B, Valentin-Hansen P, Overgaard M 2010 Translational regulation of gene expression by an anaerobically induced small non-coding RNA in Escherichia coli. J Biol Chem 285:10690–10702. doi:10.1074/jbc.M109.089755. 20075074 PMC2856277

[39] Clements A, Young JC, Constantinou N, Frankel G 2012 Infection strategies of enteric pathogenic Escherichia coli. Gut Microbes 3:71–87. doi:10.4161/gmic.19182. 22555463 PMC3370951

[40] Prokhorenko I, Zubova S, Kabanov D, Voloshina E, Grachev S 2012 Toll-like receptor 4 in phagocytosis of Escherichia coli by endotoxin-activated human neutrophils in whole blood. Crit Care 16:P80. doi:10.1186/cc11767.

[41] Agramonte-Hevia J, González-Arenas A, Barrera D, Velasco-Velázquez M 2002 Gram-negative bacteria and phagocytic cell interaction mediated by complement receptor 3. FEMS Immunol Med Microbiol 34:255–266. doi:10.1111/j.1574-695X.2002.tb00640.x. 12443825

[42] Miller SI, Ernst RK, Bader MW 2005 LPS, TLR4 and infectious disease diversity. Nat Rev Microbiol 3:36–46. doi:10.1038/nrmicro1068. 15608698

[43] Neal MD, Leaphart C, Levy R, Prince J, Billiar TR, Watkins S, Li J, Cetin S, Ford H, Schreiber A, Hackam DJ 2006 Enterocyte TLR4 mediates phagocytosis and translocation of bacteria across the intestinal barrier. J Immunol 176:3070–3079. doi:10.4049/jimmunol.176.5.3070. 16493066

[44] Wu Z, Qin W, Wu S, Zhu G, Bao W, Wu S 2016 Identification of microRNAs regulating Escherichia coli F18 infection in Meishan weaned piglets. Biol Direct 11:59. doi:10.1186/s13062-016-0160-3. 27809935 PMC5093996

[45] Luoreng Z-M, Wang X-P, Mei C-G, Zan L-S 2018 Expression profiling of peripheral blood miRNA using RNAseq technology in dairy cows with Escherichia coli-induced mastitis. Sci Rep 8:12693. doi:10.1038/s41598-018-30518-2. 30140010 PMC6107498

[46] Nguyen HTT, Dalmasso G, Müller S, Carrière J, Seibold F, Darfeuille–Michaud A 2014 Crohn’s disease-associated adherent invasive Escherichia coli modulate levels of microRNAs in intestinal epithelial cells to reduce autophagy. Gastroenterology 146:508–519. doi:10.1053/j.gastro.2013.10.021. 24148619

[47] Guo Z, Cai X, Guo X, Xu Y, Gong J, Li Y, Zhu W 2018 Let-7b ameliorates Crohn’s disease-associated adherent-invasive E coli induced intestinal inflammation via modulating Toll-like receptor 4 expression in intestinal epithelial cells. Biochem Pharmacol 156:196–203. doi:10.1016/j.bcp.2018.08.029. 30142321

[48] Maudet C, Mano M, Eulalio A 2014 MicroRNAs in the interaction between host and bacterial pathogens. FEBS Lett 588:4140–4147. doi:10.1016/j.febslet.2014.08.002. 25128459

[49] Hsieh C-H, Rau C-S, Jeng J, Chen Y-C, Lu T-H, Wu C-J, Wu Y-C, Tzeng S-L, Yang J 2012 Whole blood-derived microRNA signatures in mice exposed to lipopolysaccharides. J Biomed Sci 19:69. doi:10.1186/1423-0127-19-69. 22849760 PMC3419134

[50] Gagnière J, Bonnin V, Jarrousse A-S, Cardamone E, Agus A, Uhrhammer N, Sauvanet P, Déchelotte P, Barnich N, Bonnet R, Pezet D, Bonnet M 2017 Interactions between microsatellite instability and human gut colonization by Escherichia coli in colorectal cancer. Clin Sci (Lond) 131:471–485. doi:10.1042/CS20160876. 28093453

[51] Wilson MR, Jiang Y, Villalta PW, Stornetta A, Boudreau PD, Carrá A, Brennan CA, Chun E, Ngo L, Samson LD, Engelward BP, Garrett WS, Balbo S, Balskus EP 2019 The human gut bacterial genotoxin colibactin alkylates DNA. Science 363:eaar7785. doi:10.1126/science.aar7785. 30765538 PMC6407708

[52] Faïs T, Delmas J, Barnich N, Bonnet R, Dalmasso G 2018 Colibactin: more than a new bacterial toxin. Toxins 10:151. doi:10.3390/toxins10040151. PMC5923317 29642622

[53] Janssens Y, Nielandt J, Bronselaer A, Debunne N, Verbeke F, Wynendaele E, Van Immerseel F, Vandewynckel Y-P, De Tré G, De Spiegeleer B 2018 Disbiome database: linking the microbiome to disease. BMC Microbiol 18:50. doi:10.1186/s12866-018-1197-5. 29866037 PMC5987391

[54] Sridhar J, Gunasekaran P 2013 Computational small RNA prediction in bacteria. Bioinform Biol Insights 7:83–95. doi:10.4137/BBI.S11213. 23516022 PMC3596055

[55] Hör J, Gorski SA, Vogel J 2018 Bacterial RNA biology on a genome scale. Mol Cell 70:785–799. doi:10.1016/j.molcel.2017.12.023. 29358079

[56] Guthrie L, Gupta S, Daily J, Kelly L 2017 Human microbiome signatures of differential colorectal cancer drug metabolism. NPJ Biofilms Microbiomes 3:27. doi:10.1038/s41522-017-0034-1. 29104759 PMC5665930

[57] Zitvogel L, Ma Y, Raoult D, Kroemer G, Gajewski TF 2018 The microbiome in cancer immunotherapy: diagnostic tools and therapeutic strategies. Science 359:1366–1370. doi:10.1126/science.aar6918. 29567708

[58] Martin M 2011 Cutadapt removes adapter sequences from high-throughput sequencing reads. EMBnet J 17:10. doi:10.14806/ej.17.1.200.

[59] Kozomara A, Griffiths-Jones S 2014 miRBase: annotating high confidence microRNAs using deep sequencing data. Nucleic Acids Res 42:D68–D73. doi:10.1093/nar/gkt1181. 24275495 PMC3965103

[60] Zhang P, Si X, Skogerbø G, Wang J, Cui D, Li Y, Sun X, Liu L, Sun B, Chen R, He S, Huang D-W 2014 piRBase: a web resource assisting piRNA functional study. Database (Oxford) 2014:bau110. doi:10.1093/database/bau110. 25425034 PMC4243270

[61] Leung YY, Kuksa PP, Amlie-Wolf A, Valladares O, Ungar LH, Kannan S, Gregory BD, Wang L-S 2016 DASHR: database of small human noncoding RNAs. Nucleic Acids Res 44:D216–D222. doi:10.1093/nar/gkv1188. 26553799 PMC4702848

[62] Li H, Durbin R 2009 Fast and accurate short read alignment with Burrows-Wheeler transform. Bioinformatics 25:1754–1760. doi:10.1093/bioinformatics/btp324. 19451168 PMC2705234

[63] Love MI, Huber W, Anders S 2014 Moderated estimation of fold change and dispersion for RNA-seq data with DESeq2. Genome Biol 15:550. doi:10.1186/s13059-014-0550-8. 25516281 PMC4302049

[64] Xie B, Ding Q, Han H, Wu D 2013 miRCancer: a microRNA-cancer association database constructed by text mining on literature. Bioinformatics 29:638–644. doi:10.1093/bioinformatics/btt014. 23325619

[65] Wood DE, Salzberg SL 2014 Kraken: ultrafast metagenomic sequence classification using exact alignments. Genome Biol 15:R46. doi:10.1186/gb-2014-15-3-r46. 24580807 PMC4053813

[66] The RNAcentral Consortium. 2019 RNAcentral: a hub of information for non-coding RNA sequences. Nucleic Acids Res 47:D1250–D1251. doi:10.1093/nar/gky1206. 30535383 PMC6323998

[67] Lorenz R, Bernhart SH, zu Siederdissen CH, Tafer H, Flamm C, Stadler PF, Hofacker IL 2011 ViennaRNA package 2.0. Algorithms Mol Biol 6:26. doi:10.1186/1748-7188-6-26. 22115189 PMC3319429

[68] Bonnal RJP, Rossi RL, Carpi D, Ranzani V, Abrignani S, Pagani M 2015 miRiadne: a web tool for consistent integration of miRNA nomenclature. Nucleic Acids Res 43:W487–W492. doi:10.1093/nar/gkv381. 25897123 PMC4489305

[69] Mjelle R, Sjursen W, Thommesen L, Sætrom P, Hofsli E 2019 Small RNA expression from viruses, bacteria and human miRNAs in colon cancer tissue and its association with microsatellite instability and tumor location. BMC Cancer 19:161. doi:10.1186/s12885-019-5330-0. PMC6381638 30786859

[70] Neerincx M, Sie DL, van de Wiel MA, van Grieken NC, Burggraaf JD, Dekker H, Eijk PP, Ylstra B, Verhoef C, Meijer GA, Buffart TE, Verheul HM 2015 MiR expression profiles of paired primary colorectal cancer and metastases by next-generation sequencing. Oncogene 4:e170. doi:10.1038/oncsis.2015.29. PMC4632090 26436952

[71] Sun G, Cheng YW, Lai L, Huang TC, Wang J, Wu X, Wang Y, Huang Y, Wang J, Zhang K, Hu S, Yang JR, Yen Y 2016 Signature miRNAs in colorectal cancers were revealed using a bias reduction small RNA deep sequencing protocol. Oncotarget 7:3857–3872. doi:10.18632/oncotarget.6460. 26646696 PMC4826175

