# Correction for Tarallo et al., “Altered Fecal Small RNA Profiles in Colorectal Cancer Reflect Gut Microbiome Composition in Stool Samples”

**DOI:** 10.1128/mSystems.00072-20

**Published:** 2020-02-18

**Authors:** Sonia Tarallo, Giulio Ferrero, Gaetano Gallo, Antonio Francavilla, Giuseppe Clerico, Alberto Realis Luc, Paolo Manghi, Andrew Maltez Thomas, Paolo Vineis, Nicola Segata, Barbara Pardini, Alessio Naccarati, Francesca Cordero

**Affiliations:** a Italian Institute for Genomic Medicine (IIGM), Turin, Italy; b Department of Computer Science, University of Turin, Turin, Italy; c Department of Surgical and Medical Sciences, University of Catanzaro, Catanzaro, Italy; d Department of Colorectal Surgery, Clinica S. Rita, Vercelli, Italy; e Department CIBIO, University of Trento, Trento, Italy; f Imperial College, London, United Kingdom; g Department of Medical Sciences, University of Turin, Turin, Italy; h Department of Molecular Biology of Cancer, Institute of Experimental Medicine, Prague, Czech Republic

## AUTHOR CORRECTION

Volume 4, no. 5, e00289-19, 2019, https://doi.org/10.1128/mSystems.00289-19. The following text corrections are necessary.

Page 2, third line from the bottom, the paragraph should read: “At the phylum level, the abundances of *Proteobacteria* and *Verrucomicrobia* in adenoma patients were significantly different from those seen in the healthy group. In particular, *Verrucomicrobia* showed the lowest abundance, whereas *Proteobacteria* showed intermediate abundance in the adenoma group (see Table S1A). *Proteobacteria* was the most significantly abundant phylum in the carcinoma group compared with both the healthy and adenoma groups, while *Firmicutes* abundance significantly decreased from the healthy group to the carcinoma group (Fig. 1A; see also Table S1A).”

Page 5, Fig. 2 legend should read: “(A) Flow chart summarizing the analyses performed to identify and analyze the sRNA-Seq reads assigned to microbial genomes and bsRNAs. WMS, whole-metagenome sequencing. (B) Stacked bar plots reporting the relative abundances of bacterial phyla detected using whole-metagenome sequencing (top) and small RNA sequencing (sRNA-Seq; bottom) data, respectively. (C) Bar plot reporting the Pearson correlation coefficient (*r*) computed from comparisons between metagenomic and sRNA-Seq data for each phylum. (D) Heat map representing the log_10_ numbers of reads assigned to bsRNAs from the Bacteria Small RNA Database (BSRD). Only annotations that were significantly different (adjusted *P* value of <0.05) between healthy, adenoma, and CRC groups are shown. *P.aeu*, P. aeruginosa. (E) Heat map reporting the log_2_ ratios of relative abundances between bsRNAs and bacterial DNA profiles. Only ratios of bacterial species with median values greater than 1 are reported.”

Page 5, fourth line from the bottom, the text should read: “The most correlated bacterial phyla were *Spirochaetes* (*r *= 0.902), *Proteobacteria* (*r *= 0.786), and *Fusobacteria* (*r *= 0.646) (Fig. 2C). At the species level, Porphyromonas asaccharolytica was characterized by the highest correlation (*r *= 0.999; adjusted *P* value of <0.0001), while F. nucleatum (*r *= 0.990; adjusted *P* value of <0.0001) and E. coli (*r *= 0.632; adjusted *P* value of <0.0001) were among the 15 most highly correlated species (see Table S4C).”

Page 9, sixth line from the top, the text should read: “In our analyses, the *Firmicutes* abundances characterizing cancer samples were significantly different from those characterizing the healthy group. Interestingly, the *Verrucomicrobia* phylum, characterized by a significant decrease in abundance in the adenoma samples, may represent a potential candidate biomarker for precancer lesions.”

